# Inequalities in public health care delivery in Zambia

**DOI:** 10.1186/1475-9276-13-24

**Published:** 2014-03-19

**Authors:** Jane Phiri, John E Ataguba

**Affiliations:** 1 Health Economics Unit, School of Public Health and Family Medicine, University of Cape Town Observatory, Cape Town 7925, South Africa

**Keywords:** Health inequality, Health inequity, Public health care, Zambia

## Abstract

**Background:**

Access to adequate health services that is of acceptable quality is important in the move towards universal health coverage. However, previous studies have revealed inequities in health care utilisation in the favour of the rich. Further, those with the greatest need for health services are not getting a fair share. In Zambia, though equity in access is extolled in government documents, there is evidence suggesting that those needing health services are not receiving their fair share. This study seeks therefore, to assess if socioeconomic related inequalities/inequities in public health service utilisation in Zambia still persist.

**Methods:**

The 2010 nationally representative Zambia Living Conditions and Monitoring Survey data are used. Inequality is assessed using concentration curves and concentrations indices while inequity is assessed using a horizontal equity index: an index of inequity across socioeconomic status groups, based on standardizing health service utilisation for health care need. Public health services considered include public health post visits, public clinic visits, public hospital visits and total public facility visits.

**Results:**

There is evidence of pro-poor inequality in public primary health care utilisation but a pro-rich inequality in hospital visits. The concentration indices for public health post visits and public clinic visits are −0.28 and −0.09 respectively while that of public hospitals is 0.06. After controlling for need, the pro-poor distribution is maintained at primary facilities and with a pro-rich distribution at hospitals. The horizontal equity indices for health post and clinic are estimated at −0.23 and −0.04 respectively while that of public hospitals is estimated at 0.11. A pro-rich inequity is observed when all the public facilities are combined (horizontal equity index = 0.01) though statistically insignificant.

**Conclusion:**

The results of the paper point to areas of focus in ensuring equitable access to health services especially for the poor and needy. This includes strengthening primary facilities that serve the poor and reducing access barriers to ensure that health care utilisation at higher-level facilities is distributed in accordance with need for it. These initiatives may well reduce the observed inequities and accelerate the move towards universal health coverage in Zambia.

## Introduction

The positive relationship between economic growth and health has increased the interests of researchers, governments, decision makers and international organisations in inequities in health and health service utilisation and how to address them [1,2]. Access to adequate health services that is of acceptable quality is also regarded as important in the move towards universal health coverage [3]. However, in many countries, especially the developing countries, there is evidence of wide inequalities in the utilisation of health services as well as the presence of the inverse care law; those with the greatest need for health services are not getting a fair share from health services [3-9]. As a result, these inequities contribute to and intensify disparities in health and quality of life [10,11]. In the literature, in relation to health service utilisation, inequality exists when there are differences in utilisation by socioeconomic status [12] while inequity occurs when utilisation is unequal and unfair for everyone after considering the differences in needs [13]. Equity could be horizontal or vertical. Horizontal equity means equal treatment for individuals with equal need while vertical equity means unequal treatment for individuals with different health needs [14]. Horizontal equity is often seen as the most relevant for assessing health service delivery.

In Zambia, health services are largely financed from public tax, donor community grants and direct payments by households and are provided by the government, private not-for-profit and private for-profit providers [15]. The delivery of government services is organised at three broad levels of care: tertiary level, comprising tertiary teaching hospitals; secondary level, comprising provincial/general hospitals and district hospitals; and the primary level, consisting of health centres and health posts [16]. In Zambia, just like in many other countries, equity in the distribution of health care utilisation is recognised to be important in developing public policies aimed at reducing poverty and fostering development. In this regard, the country’s Vision 2030 asserts the right of equality in access to and use of good quality health care for all regardless of socioeconomic status [17]. However, inequality remains high and there is evidence suggesting the existence of the inverse care law [16,18]. The growing inequalities can be traced to the period when Structural Adjustment Programs (SAPs) were introduced in 1991 that led to the imposition of user fees. This also led to a decrease in health service utilisation especially amongst the poor [19,20]. In response, the Zambian government initiated pro-poor policies and initiatives to increase health service utilisation, improve health outcomes, and respond to the people’s needs whilst guaranteeing financial risk protection [21]. The entire health system was decentralised in 1995 and user fees were abolished initially at all rural facilities in 2006 but later rolled out to all primary facilities throughout the country in 2007 [22,23]. The result was an improvement in some health indicators and health service utilisation especially at primary facilities [23,24].

Recently, while some studies have examined the extent of inequalities, they mainly focus on specific health outcomes and health interventions [25,26]. These studies are nonetheless important but more is required in order to appraise the entire health system. Only two studies in Zambia have explicitly explored inequalities/inequities in health care utilisation. Both revealed pro-rich inequalities [18,27]. Bonfer et al., found evidence of a slightly pro-rich bias comparing health care use and health need across socioeconomic quintiles. The horizontal equity index of general health care use was 0.01 [18]. After adjusting for self-perceived health need, Zyaambo et al. found that in rural Zambia, individuals in the highest wealth quintile were three times more likely to use health services than those in the lowest wealth quintile [27].

These studies were undertaken not too long after the pro-poor reforms were introduced. Further, they only present aggregated results which do not distinguish between facility levels. In this regard this paper seeks to provide more recent evidence, disaggregated by facility levels, and to determine whether amidst more recent health reforms inequities still persist. Specifically, the paper analyses socioeconomic inequalities and inequities in the utilisation of public health service in Zambia.

## Methods

### Data source

Data are obtained from the 2010 Zambian Living Conditions Monitoring Survey (LCMS), which is popularly known as the Indicator Monitoring Survey. This is a nationally representative survey that aims to monitor the levels of development and poverty in the country [28]. It is conducted by the Central Statistical Office (CSO) between January and April of 2010 and it used a two-staged stratified cluster sampling strategy. The first step involved the selection of 1000 Standard Enumeration Areas (SEAs) with Probability Proportional to Size (PPS) [28]. Next, approximately 20,000 households are systematically selected across the SEAs, which comprised both rural and urban locations, and the nine provinces [28]. With a household response rate of about 98%, the complete dataset contains 19,398 households (i.e., 102,882 individuals). In terms of content, the survey includes a wide range of information including health, living conditions of individuals and households, economic activities as well as demographic characteristics.

### Measuring socioeconomic related status

Socioeconomic status is assessed using a proxy (i.e. household consumption expenditure). Consumption expenditure is considered a more reliable measure as compared to both income and asset index. Unlike income, it is less variable, less susceptible to being under-reported and unlike asset index, offers a better reflection of current living conditions [2,29]. Despite having its own shortcomings consumption expenditure is a better method to use in situations where an organised labour market is lacking [2]. Household consumption expenditure is computed using the following categories; food, transport, utilities, housing, beverages and tobacco, durable and non-durable household goods, household produced commodities and frequently purchased services. Questions captured different recall periods. Therefore, conversion factors are applied to come up with a common reference period (i.e., annualised consumption expenditure). Household expenditure is further adjusted for household size and composition [29,30] using an adult equivalent scale (*E*), which is obtained as follows:

(1) $$E={\left(A+\mathrm{\alpha K}\right)}^{\beta }$$

where *A* = number of adults (at least 16 years old), *K* = number of children (below 16 years), α is the child adjustment factor which is a measure of the weight accorded to children relative to adults and β is elasticity, capturing economies of scale [30]. In many cases, the choice of α and β may be subjective. The recommend values are in the range of 0.3 to 0.5 for α and 0.75 to 1.0 for β. This is because food accounts for a large proportion of total consumption consequently; economies of scale are relatively limited [30]. Based on previous studies in Africa, α and β are set at 0.5 and 0.75 respectively [30-32]. Sensitivity analysis is however performed using the extreme values of 0.3 and 1.0 for α and β respectively.

### Measuring utilisation

Utilisation is measured by self-reported use or visits to public health facilities. Respondents are asked to state whether they visited a health facility or not and which kind of facility they visited if at all they did. Facility levels considered include public health posts, public clinics and public hospitals. Gender differences in health care utilisation are well established. It is commonly revealed in studies that deal with morbidity/illness and health care utilisation that women generally report more ill health symptoms, report worse health status and are also more likely to seek medical attention [7]. Further, older persons are more susceptible to being ill and ideally ought to use more health care services [7]. In addition, both age and sex are related to socioeconomic status [2]. This means that age and sex are confounding variables. It is for this reason that health care utilisation in this paper is adjusted for age and sex. Standardisation does not build a casual, or structural, model of health care utilisation determination but to provide a more distinguished relationship between a health care utilisation variable and socioeconomic status [29]. Such adjustment could be done using the direct or indirect standardisation. This study uses indirect standardisation because standardising using the direct approach always requires the use of grouped data which makes the estimated inequality measure highly dependent on the number of socioeconomic groups [29]. When employing the indirect standardisation method, the first step involves the estimation of the predicated health care utilisation ${\hat{h}}_{i}^{X}$ (one that incorporates age and sex)

(2) $${\hat{h}}_{i}^{X}=\alpha +{\hat{\beta }}_{j}x_{\mathrm{ij}}$$

where *x*_*ij*_ are the confounding variables (i.e., age and sex).

Finally, age and sex standardised health care utilisation ${\hat{h}}_{i}^{\mathrm{IS}}$ is obtained as:

(3) $${\hat{h}}_{i}^{\mathrm{IS}}=h_{i}+{\hat{h}}_{i}^{X}+\mu$$

where *hi* is the actual utilisation value, ${\hat{h}}_{i}^{X}$ is the predicted value obtained from equation (2) and μ is the mean of the health care utilisation variable.

### Measuring need

In this paper, the variable ‘need’ is based on self-reported health. This represents respondents who reported being ill and/or injured in the 2 weeks prior to the survey and/or declared being continuously ill in the previous three months and/or facing difficulties in performing normal tasks^a^. Merely relying on an individual’s declaration of their illness is considered by many to be an ineffective measure of need for health care because it is a very subjective measure with a tendency for being under-reported among the poor [10,18]. However, these measures have been used in recent studies in Africa [3,9].

### Measuring inequality in health care utilisation

**Figure 1:**
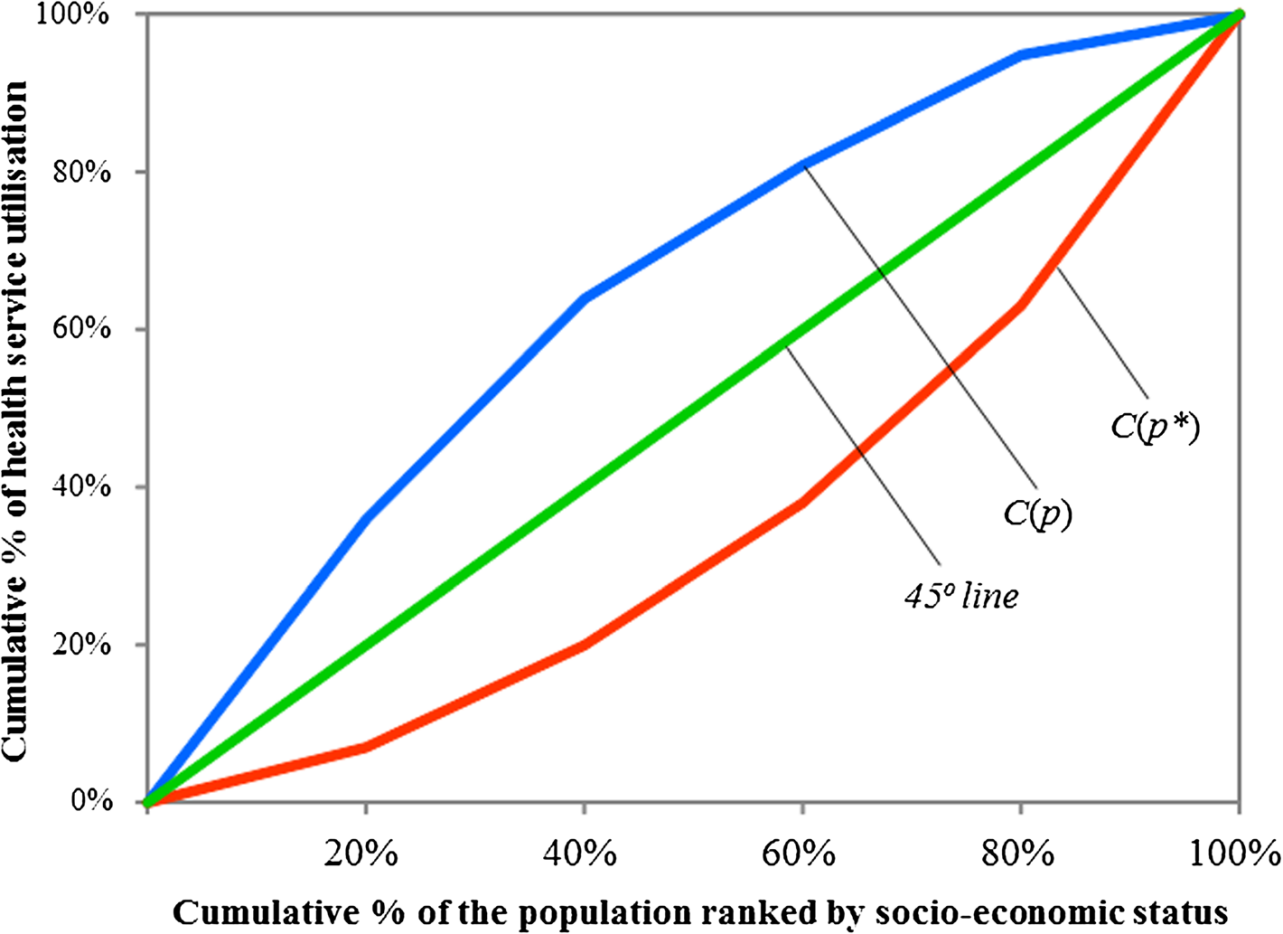
Concentration curve for health care utilisation illustrated.

Concentration curves and indices are used to examine how pro-poor/pro-rich the distribution of public health care utilisation is. A concentration curve plots the cumulative share of the health variable (i.e., age-sex standardised utilisation) against the cumulative shares of households in the population ranked from poorest to richest [12,33]. As shown in Figure 1, if the concentration curve *C*(*p*) lies above the 45 degree line (i.e., the line of equality) then health care utilisation is concentrated among the poor whilst if the concentration curve *C*(p*) falls below the line of equality then the opposite is the case [33]. Further, if the concentration curve matches with the line of equality (a case of proportionality) then health care utilisation is equally distributed across groups.

Because concentration curves are estimated from survey data with sampling variability, formal statistical tests of dominance between concentration curves and the line of equality become necessary [29]. This involves testing whether the observed pro-poor or pro-rich distribution is not statistically equivalent to a case of proportionality^b^.

Concentration indices (CIs) are obtained from the associated concentration curves as twice the area between a concentration curve, say *C*(*p*), and the line of equality. It takes on values between −1 (when the population’s health care utilisation is concentrated among the poor) and +1 (when the population’s health care utilisation is concentrated among the rich). Generally, a positive index signifies that the distribution of utilisation is higher among the richer groups while a negative index indicates the opposite.

In this paper, the age-sex standardised concentration indices (*CI*_*IS*_) and their standard errors are obtained [34] as:

(4) $$CI_{\mathrm{IS}}=1-\left({{\hat{\xi }}_{H}/\hat{\mu }}_{H}\right)$$

where the vector of per adult equivalent incomes **x** = [*x*_1_,  *x*_2_ ⋯,  *x*_*n*_] is the ranking variable such that $x_{1}\geq x_{2}\geq \cdots \geq x_{n-1}\geq x_{n};\;{\hat{\xi }}_{H}=\sum_{i=1}^{n}\left(\left({\left(V_{i}\right)}^{2}-{\left(V_{i+1}\right)}^{2}\right)/{\left(V_{1}\right)}^{2}\right)h_{i};\;h_{i}$is the indirectly standardised health care utilisation for individual $i;\;{\hat{\mu }}_{H}$ is the mean of $h_{i};\;V_{i}=\sum_{j=i}^{n}w_{j};$ and the vector **w** = *w*_1_,  *w*_2_, ⋯,  *w*_*n*_ represents the appropriate sampling weights.

It has been revealed that the lower and upper limits of the concentration index for a dichotomous variable are not −1 and 1 respectively but lie between μ – 1 and 1 – μ for large samples, where μ is the mean of the variable [35]. There has been some debate concerning how best to normalise this. Wagstaff recommends a normalisation process that involves diving the concentration index by 1 – μ. Erreygers, criticises Wagstaff’s method on a number of respects including the fact that it does not possess the mirror effect property (i.e., inequality in use being equal to inequality in non-use) [36]. Erreygers further criticises Wagsataff’s approach and suggests that it “blow(s) up the levels of measured inequality for distributions with either high or low means” [37] p.523. Wagstaff’s approach exhibits little variation between the normalised index and the ordinary index and the ordering of inequality also remains the same for both measures [38]. In addition, Erreygers’ normalised concentration index can be obtained by scaling the Wagstaff’s normalised index in the case of a binary variable [38]. Therefore in this paper, the Wagstaff’s normalisation is used.

The normalised concentration indices (*C*_*H*_) are computed as follows [35]:

(5) $$C_{H}=CI_{\mathrm{IS}}/\left(1-\mu^{\mathrm{IS}}\right)$$

with *CI*_IS_ and μ^IS^ as previously defined.

### Measuring inequity in utilisation

The concept of horizontal equity in the health care utilisation requires that persons with equal need use health care services equally [14]. Based on this principle, this paper measures the degree of inequity in health care delivery using a common approach previously proposed [33]. Under this approach, comparing each socioeconomic group’s share of need with its share of health service use assesses the extent of horizontal inequity. The index of inequity (HI) that is used for this purpose is defined as two times the area between the need and health service use concentration curves [33]. A positive *HI* value signifies a pro-rich inequity and a negative value signifies a pro-poor inequity and a value of zero shows that health care utilisation and need are proportionally distributed across the socioeconomic distribution. The horizontal equity index is mathematically obtained by subtracting the concentration index for need from the concentration index of health service utilisation.

Need is standardised analogously as health care utilisation for sex and age. Also, the age-sex standardised concentration index for need (*CI*_*N*_) and the normalised need concentration index (*C*_*N*_) are obtained in a similar way to health care utilisation.

The normalised/age-sex standardised horizontal equity index is numerically computed as the difference between the normalised health service utilisation index *C*_*H*_ and the normalised need concentration index *C*_*N*_.

(6) $$\mathrm{HI}=C_{H}-C_{N}$$

All analyses are performed in Stata® version 12 taking into account the sample design for the LCMS.

## Results

Overall, about 14% of the population reported needing health services (Table 1). Higher proportions of poorer quintiles reported being ill/injured and/or having been continuously ill for 3 months prior to the interview and/or facing limitations in usual activities. Illness/injuries contributed the most to total health care need compared with continuous illness and functional limitation. As seen from Table 1 there are marked differences in average facility utilisation. Clinics accounted for 56% of all public facility visits while hospitals accounted for 42% and health centre utilisation accounted for just 2%.

**Table 1:** Distribution of need variables and facility use in Zambia, 2010

	**Q1 (poorest)**	**Q2**	**Q3**	**Q4**	**Q5 (richest)**	**P-value**	**Total**
	**Percent (%)**						
**Need variables**							
Ill/injured	12.80	14.56	14.71	13.59	11.30	p < 0.01	13.17
Continuous illness	1.43	1.43	1.61	1.68	1.15	p < 0.01	1.44
Functional incapability	1.00	0.97	1.13	1.11	0.75	p < 0.01	0.97
Need (ill/injured, continuous illness, Functional incapability)	13.56	15.19	15.52	14.45	11.87	p < 0.01	13.88
**Facility utilisation**							
Health centres	0.30	0.25	0.22	0.08	0.06	p < 0.01	0.16
Clinics	4.71	4.67	4.54	4.07	2.47	p < 0.01	3.88
Hospitals	2.10	2.90	3.16	3.34	2.93	p < 0.01	2.94
All government facilities	7.11	7.82	7.92	7.49	5.45	p < 0.01	6.97

### Inequality and inequity in health care utilisation

**Figure 2:**
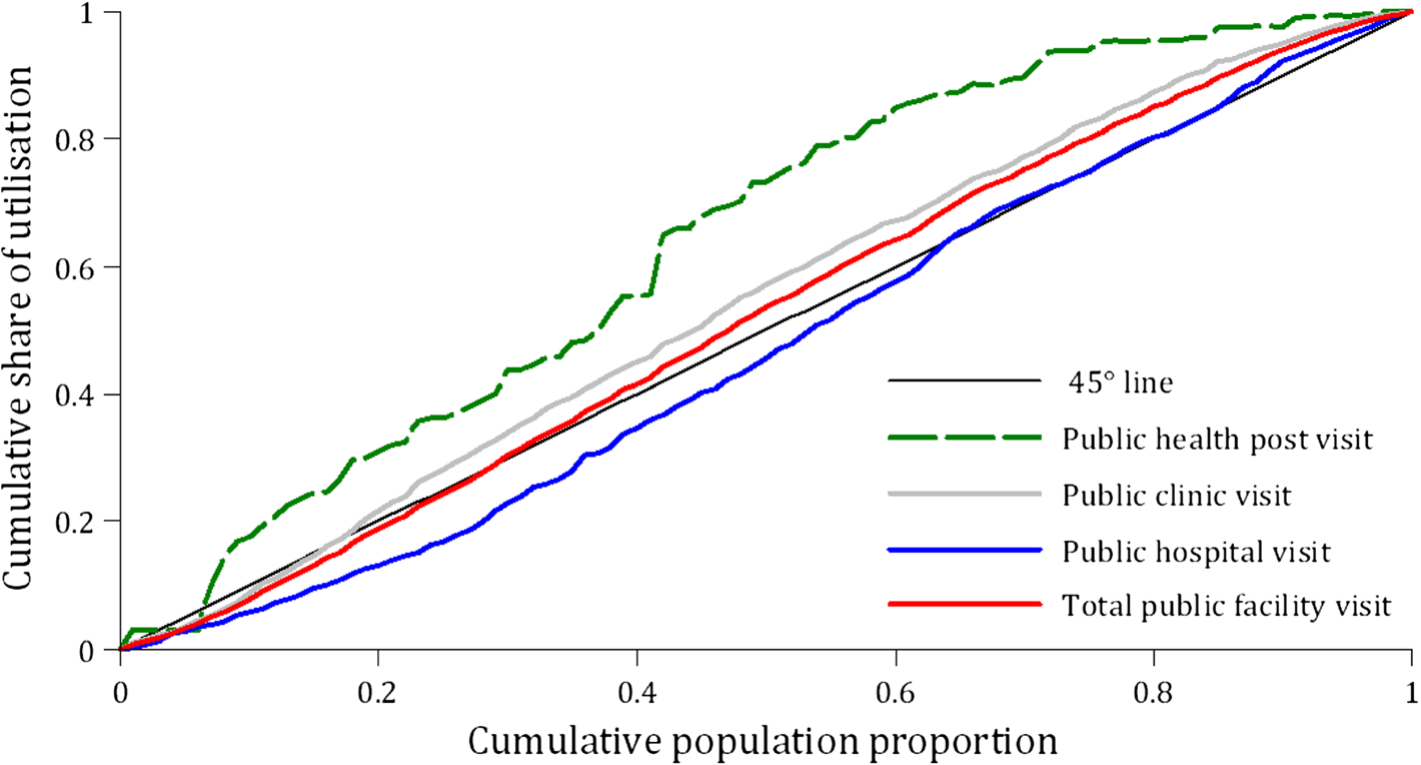
Concentration curves for health care utilisation in Zambia, 2010.

The graph shown in Figure 2, where the 45-degree line (i.e., the line of equality) and the concentration curves for health service utilisation are plotted, give a visual sense of the presence of inequalities.

Utilisation of public health posts is to the advantage of the poor because the concentration curve lies above the line of equality. The concentration curves for public clinic visits and all public facility visits mostly lie above the line of equality implying that utilisation is to the advantage of the poor. On the contrary, the concentration curve for public hospital utilisation mostly lies below the line of equality. This is indicative of a pro-rich utilisation pattern. These results are also confirmed in Table 2, which shows statistically significant negative concentration indices for public health post visits, public clinic visits and all public facility visits. The concentration index for public hospital visits is positive and statistically significant at the 5% level.

**Table 2:** Socioeconomic related inequality and inequity in public health care utilisation in Zambia, 2010

**Facility**	**Concentration index**	**Horizontal equity index**
**Public health post visit**	−0.2795** (0.0602)	−0.2295** (0.0649)
**Public clinic visit**	−0.0915** (0.0222)	−0.0415* (0.0203)
**Public hospital visit**	0.0590* (0.0230)	0.1091** (0.0290)
**Total public facility visit**	−0.0447** (0.0164)	0.0054 (0.0161)
***Need***	−0.0501** (0.0244)	

*Note*: *p < 0.05; **p < 0.01. Standard errors in parenthesis.

When need is adjusted for, as shown in Table 2, similar results are obtained for the horizontal equity indices. The significantly negative horizontal equity indices for public health post visits and public clinic visits indicate unequal utilisation for given need. This distribution is to the advantage of the worse-off. While horizontal inequity appears to favour the poor for primary public facilities (i.e., health posts and clinics), the magnitude of inequity varies, with a stronger pro-poor bias observed at health posts. Similarly, when pro-rich inequality in public hospital visits is adjusted for health care need, it resulted in a pro-rich horizontal inequity distribution. On the other hand, despite the pro-poor inequalities at all public facilities, when adjusted for need, a low pro-rich inequity is detected though not statistically significant at conventional levels.

**Figure 3:**
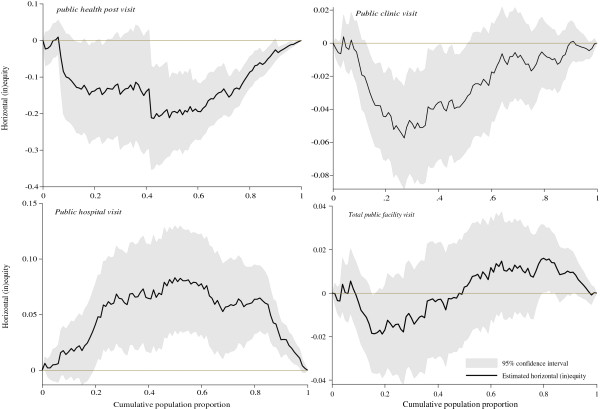
Horizontal inequity curves for health care utilisation in Zambia, 2010.

The horizontal inequity index is also presented graphically in Figure 3. The same conclusions are arrived at – there is significant horizontal equity in visits to lower level facilities that favours the poor and needy while significant pro-rich inequity exists in visits to public hospitals.

The results presented in Table 2, and in Figures 2 and 3 are based on the assumption that α and β (i.e., the equivalence scale coefficients) are 0.5 and 0.75 respectively. When α and β are set at 0.3 and 1.0 respectively (i.e., the extreme recommended values), the actual concentration indices and horizontal indices values change but the decision rules of either a pro-poor or pro-rich distribution remain identical to those presented.

## Discussion

This paper shows that the rich visit public health facilities more, especially public hospitals. Besides having lower need, the rich have higher visits to public health facilities in total. These results suggest the presence of the inverse care law, which states that poor people, who are also confronted with higher ‘need’, use fewer health care services [8]. This is in line with the findings of previous studies that revealed distributions in general health care that favoured the rich [18,27]. Similarly, pro-rich distributions have been reported in studies from other developing countries [6,7,39].

When stratified by levels of care, the poor have greater use of primary facilities (i.e., health posts and clinics) in relation to need. This pro-poor distribution with regard to these facilities is similar to the results reported elsewhere that primary facilities offer the best option for reaching the poor [40,41]. In the same vein, primary facility use has been found to disproportionally benefit the poor in other countries outside Africa including Taiwan, South Korea and Israel [39,42]. Other studies, though not explicitly focusing on equity in health care utilisation, but on monetary benefits of utilising health care find similar results. In Kenya, South Africa and Tanzania for instance, the poor derive more benefits from using public primary facilities [9,43,44].

In the case of Zambia, considering the removal of user fees at primary health facilities, it may not be unrealistic to see a pro-poor distribution. It is possible that the pro-poor inequities in primary health care use could be attributed to the reduced physical access barriers, as the numbers of health posts and clinics have been increased with most of them located within communities [16].

On the other hand, public hospital utilisation is highly concentrated among the rich when need is taken into account. Similar results have been reported in South Africa [41] even though these authors did not distinguish between public and private hospitals. Comparable results in terms of monetary benefits are also found in some African countries, where the benefits of using public hospitals are highly concentrated among the well-off that have less need [9,43,44].

Previous studies on health service utilisation in Zambia have revealed that utilisation is concentrated among individuals of higher socioeconomic status [18,27]. These studies did not stratify analysis by the levels of care and by ownership structure (i.e., public or private funded). These studies were also undertaken not too long after the pro-poor reforms (i.e., user fee removal) were put in place. While these previous studies are enlightening, the current paper provides more recent evidence, considering that more pro-poor health initiatives such as building of more health facilities within rural communities occurred long after the previous studies were conducted. Further, this paper stratifies the analysis by levels of care, so as to have a clearer picture of the presence and extent of inequity for the different facility levels.

The main strength of this paper is its ability to specifically disaggregate the analysis by different health care facility levels, which has not really been a main focus of health care utilisation inequity studies done in many African countries. This stratification revealed that the pro-rich distribution of overall public health care services is due to the pro-rich distribution of higher-level facilities, particularly hospitals. In addition, this study standardises the utilisation and need variables for age and sex variations, an approach that is not taken by many previous studies. The standardisation enables the estimation of a more refined description of the relationship between the two variables (health care utilisation and need) and socioeconomic status [29].

This paper has a few limitations. Firstly, like all other studies that use household survey data, the data collected from the 2010 Zambia LCMS, particularly for the variables of health service utilisation, and need measurement, are subject to respondents recall biases [45]. Further, the utilisation variables are dichotomous and do not allow for the computation of actual utilisation rates [46]. Secondly, medical determination of a more objective need for health care services is hardly a feasible task within the LCMS context. This paper uses self-reported illness and limitations in functional capabilities to measure ‘need’ for health care. It has generally been stated that socioeconomic differences affect respondent’s ability to interpret some symptoms as marking an episode of illness [18]. Respondents of different socioeconomic groups may have dissimilar evaluations about what ‘normal’ health status ought to be. It is generally noted that poorer groups are less likely to report illness by modifying their illness perception as a coping strategy to prevent them from incurring the costs associated with illness [5]. This may well be represented in the lower percentages recorded in the poorest quintile compared to the second poorest quintile in Table 1 for the need variables. Additionally, this paper does not take into consideration the quality of health care between facilities.

Based on the results, it important to note that the pro-poor public primary facility use has implications for ensuring equity in overall health care utilisation in Zambia and in the current debate around ensuring universal health coverage. Evidence of pro-poor utilisation patterns of health care is an important shift that could be used for advocating greater allocation of resources to public primary facilities that have been found to greatly benefit the poor. Further, this pro-poor distribution at primary level of care calls for initiatives to improve quality of health care services provided at these primary facilities. This will further promote the health of the poor [2]. The pro-rich results for hospital use and overall public facility visits also have some policy implications. Because the poor are faced with physical and financial barriers to access, government policies aimed at improving physical and financial accessibility to public hospitals would probably be a move in the right direction considering that such measures have been shown to yield positive results, at primary facilities in Zambia.

## Conclusion

Access to and utilisation of adequate health care services that is of acceptable quality are essential aims in the move towards universal health coverage. The results of the paper point to areas of focus in ensuring equitable access to health services especially for the poor and needy. This includes strengthening primary facilities that serve the poor and reducing access barriers to ensure that health care utilisation at higher-level facilities is distributed in accordance with need for it. Further, there is a need for further research that goes beyond just quantifying inequality/inequity, to examine what factors drive this distribution of health care utilisation within the context of Zambia.

## Endnotes

^a^The three questions in the LCMS include (a) “Have you been ill or injured in the last 2 weeks?” (b) “Have you been continuously ill for at least 3 months in the last twelve months?” and (c) “Are you able to carry out normal activities during the period of illness?”

^b^Though the paper considers the test of dominance, the results are however not shown.

## Abbreviations

LCMS: Living conditions and monitoring survey; SAPs: Structural adjustment programs.

## Competing interests

The authors declare that they have no competing interests.

## Authors’ contributions

JP was involved in the conception and design of the study, acquisition of data, data analysis, interpretation of data, drafting and revising the manuscript. JEA was involved in the conception and design of the study, data analysis and interpretation, drafting and revising the manuscript. Both authors read and approved the final manuscript.
